# Comment on: Lee J.Z.C. et al. Case report: Hepatocellular carcinoma in a patient with Pyridoxamine 5-phosphate oxidase (PNPO) deficiency undergoing pyridoxal 5-phosphate (PLP) treatment. Mol Genet Metab Rep. 2025;9;43:101224.

**DOI:** 10.1016/j.ymgmr.2025.101248

**Published:** 2025-08-26

**Authors:** P. De Liso, R. Webster, B. Plecko, F. Vigevano

**Affiliations:** a Bambino Gesù Children's Hospital, IRCCS, Management and Diagnostic Innovations & Clinical Pathways Research Area, Neurorehabilitation and Adapted Physical Activity Day Hospital, Rome, Italy; b The Children's Hospital at Westmead, T.Y. Nelson Department of Neurology and Neurosurgery and the Kids Neuroscience Centre, Sydney, Australia; c Medical University of Graz, Department of Pediatrics, Division of General Pediatrics, Graz, Austria; d San Raffaele, IRCCS, Developmental Disabilities Department, Rome, Italy

Dear Sir,

We read with great interest the case report by Lee et al. recently published in Molecular Genetics and Metabolism Reports [1], describing hepatocellular carcinoma (HCC) in a child with PNPO deficiency treated with long-term pyridoxal 5′-phosphate (PLP). However, it is worth noting that this is not the first reported case of Hepatocellular Carcinoma associated with PLP treatment in patients with PNPO deficiency.

In our recent letter published in European Journal of Paediatric Neurology, we reported two unrelated cases of PNPO deficiency who also developed liver cirrhosis followed by HCC after prolonged high-dose PLP treatment [2]. Our findings are consistent with those of Lee et al., particularly regarding the early onset of liver abnormalities, since both their patient and our patients developed liver disease between 2 and 3 years of age with a subsequent diagnosis of HCC, which occurred between 12 and 14 years in both reports.

Nevertheless, some important differences should be highlighted. While Lee et al. describe PLP therapy at a dose of 40 mg/kg/day, our patients were treated with higher doses, up to 50–100 mg/kg/day. Despite this, liver injury and HCC occurred across this dosing range, suggesting that dosage alone may not fully explain the hepatic risk or that a PLP dose of 40 mg/kg/day is not low enough to mitigate this risk.

The reason that PLP is associated with hepatotoxicity is not known. Very elevated levels of B6 vitamers (pyridoxal, pyridoxine, pyridoxamine and their breakdown product pyridoxic acid) were identified in a liver biopsy of one of the patients who subsequently developed HCC [3]. In our report, we also discussed the potential impact of PLP formulation and preparation methods, particularly extemporaneously prepared liquid formulations during infancy, as additional contributing factors. It is also possible that enteric microbial metabolism of PLP may result in hepatotoxic byproducts.

Although Lee et al. suggest dosage reduction as a primary preventive strategy, we believe that this approach, while necessary, may not be sufficient. In our view, the goal should be to use the minimum effective dose of PLP to maintain seizure control, ideally combined with antiseizure medications when appropriate, to minimize long-term hepatic risks.

In conclusion, both reports highlight the urgent need for early and rigorous liver monitoring in patients with PNPO deficiency under PLP therapy. However, we also emphasize the importance of considering formulation quality and combination treatment strategies beyond simple dose reduction. A better understanding of the underlying mechanisms by which PLP induces hepatotoxicity will be essential to improving the long-term safety of therapy in these vulnerable patients. The publication of these two studies underscores the relevance of this issue and additional similar cases may exist worldwide. It is therefore crucial to elucidate the mechanisms by which the only effective therapy can lead to such a severe adverse outcome and to investigate alternative therapeutic strategies.

## CRediT authorship contribution statement

**P. De Liso:** Conceptualization, Writing – original draft, Writing – review & editing. **R. Webster:** Writing – review & editing. **B. Plecko:** Writing – review & editing. **F. Vigevano:** Writing – review & editing.

## Declaration of competing interest

The authors declare that they have no known competing financial interests or personal relationships that could have appeared to influence the work reported in this paper.

## Data Availability

No data was used for the research described in the article.
